# Interplay of Belatacept Immunosuppression and Maribavir Antiviral Activity in Recurrent CMV Viremia: Clinical Implications and Literature Review

**DOI:** 10.3390/v17050595

**Published:** 2025-04-23

**Authors:** Imran Gani, Ahmad Mirza, Usman Baig

**Affiliations:** 1 Department of Nephrology, Hypertension and Transplant Medicine, Wellstar MCG Health, Augusta University, Augusta, GA 30912, USA; 2 Transplant Surgery Division, Wellstar MCG Health, Augusta University, Augusta, GA 30912, USA

**Keywords:** kidney transplant, immunosuppression, belatacept, cytomegalovirus, recurrent viremia, maribavir

## Abstract

Belatacept is a recombinant fusion protein used in renal transplant recipients, particularly when side effects from standard immunosuppressants occur. It offers a superior renal safety profile and is associated with better long-term renal graft outcomes. However, belatacept has been linked to atypical presentations of cytomegalovirus (CMV) infections, characterized by a prolonged and unpredictable course of viremia. We report a case involving a middle-aged African American female who developed acute kidney injury while on tacrolimus and was subsequently switched to belatacept. During treatment with belatacept, she experienced persistent and erratic CMV viremia lasting 58 weeks. The viremia showed an incomplete response to first-line antiviral therapy with valganciclovir, and the use of the novel antiviral agent maribavir also failed to achieve long-lasting viremic clearance. The resolution of the viremia was ultimately achieved only after discontinuing belatacept while continuing maribavir therapy. This case and literature review underscores the need for clinicians to remain vigilant for atypical CMV infections in renal transplant recipients treated with belatacept. If the complete clearance of viremia cannot be achieved despite the use of different antiviral agents, consideration should be given to modifying immunosuppressive therapy.

## 1. Introduction

Cytomegalovirus (CMV), a member of the herpesviridae family, is an enveloped double-stranded DNA virus often linked to significant morbidity and mortality in immunosuppressed transplant recipients. Its clinical manifestations vary widely, ranging from asymptomatic viremia to invasive disease affecting the gastrointestinal, hematological, pulmonary, central nervous, or renal systems. Although asymptomatic viremia may seem benign, it is associated with poor long-term outcomes [1]. Also, the type of immunosuppressive therapy used after renal transplantation can influence the incidence and progression of such opportunistic infections [2].

Commonly used immunosuppressants during the maintenance phase following renal transplantation include antimetabolites, calcineurin inhibitors, and glucocorticoids. Immunosuppression adjustments are often needed, particularly due to the nephrotoxicity associated with calcineurin inhibitors. One alternative used in such cases is belatacept, a T-cell co-stimulation inhibitor with several advantages, including a superior renal safety profile, better improvements in eGFR, and lower rates of long-term graft loss [3].

The common side effects of belatacept include opportunistic infections, anemia, diarrhea, hypertension, dyslipidemia, abdominal pain, and dysuria [4,5]. A phase III trial, which compared belatacept with cyclosporine, reported a similar incidence of infections, including CMV infections, across all study groups [5]. However, a recent retrospective observational study by Magua et al. has revealed an important new finding that, in high-risk patients (D+/R−), belatacept is associated with prolonged CMV viremia lasting up to 20 weeks, even with the standard antiviral treatments such as valganciclovir, foscarnet, or cidofovir [2].

We report the case of a 55-year-old African American female on belatacept infusion after a deceased donor renal transplant (DDKT) who developed CMV viremia lasting 58 weeks. Despite treatment with the novel anti-CMV agent maribavir, the viremia persisted with fluctuating levels, ultimately necessitating the discontinuation of belatacept.

## 2. Clinical Case Study

A 55-year-old African American female underwent a deceased donor kidney transplant in November 2022 for end-stage renal disease caused by focal segmental glomerulosclerosis. The donor kidney came from a 63-year-old brain-dead female donor with a witnessed out-of-hospital cardiac arrest. The donor was a smoker (>20 pack years) and had more than 10 years of hypertension history. Induction immunosuppressive therapy was initiated as per institutional protocol with antithymocyte globulin (1.5 mg/kg/day IV for 3 days), mycophenolate mofetil (750 mg, PO twice daily), and methylprednisolone (500 mg IV). This was followed by maintenance therapy, comprising mycophenolate mofetil (500 mg twice daily due to GI side effects), tacrolimus (with a goal level of 8–10 ng/mL), and prednisone (5 mg, PO once daily). The donor was CMV IgG positive (D+) and the recipient was CMV negative (R−; recipient had a negative CMV IgM and IgG during pre-transplant workup). Antimicrobial prophylaxis was initiated per protocol with valganciclovir (renally dosed 450 mg, once daily) and trimethoprim-sulfamethoxazole (80/400 mg, once daily) for six months each. During subsequent follow-up visits, the patient’s serum creatinine settled around 1.7–2.0 mg/dL.

At the 10-week follow-up visit, the patient was in her usual state of health, but routine laboratory tests revealed a sharp increase in serum creatinine to 2.8 mg/dL. Her tacrolimus trough level on the same day was 6.9 ng/mL. A transplant ultrasound did not show any hydronephrosis or transplant renal artery stenosis. Acute transplant rejection was suspected, prompting a biopsy of the transplanted kidney. At this time, serum BK virus DNA and donor-specific antibodies (DSAs) were negative. A histopathological examination of the biopsy tissue revealed changes consistent with acute kidney injury (simplification of tubular epithelium with loss of brush borders, and reactive-appearing nuclei). Arterioles showed moderate hyalinosis and arteries showed arteriosclerosis with moderate–severe intimal fibrosis. SV40 staining was negative for BK virus. Urinary tract infection and supratherapeutic tacrolimus levels were ruled out. Creatinine did not return to baseline and peaked at 3.1 mg/dL within the next five weeks. With no other cause for the persistent elevation of creatinine, and given the vascular findings on biopsy, vasoconstriction-related functional tacrolimus renal toxicity was suspected, and the patient was transitioned to belatacept (5 mg/kg infusion every 28 days) while tapering off tacrolimus in February 2023. The patient’s Epstein-Barr Virus serological panel as part of her pre-transplant workup was suggestive of past EBV infection (EBV IgG +). Belatacept infusions were continued, and serum creatinine steadily declined to 2.2 mg/dL by May 2023. By this time, CMV prophylaxis had been completed.

**Figure 1:**
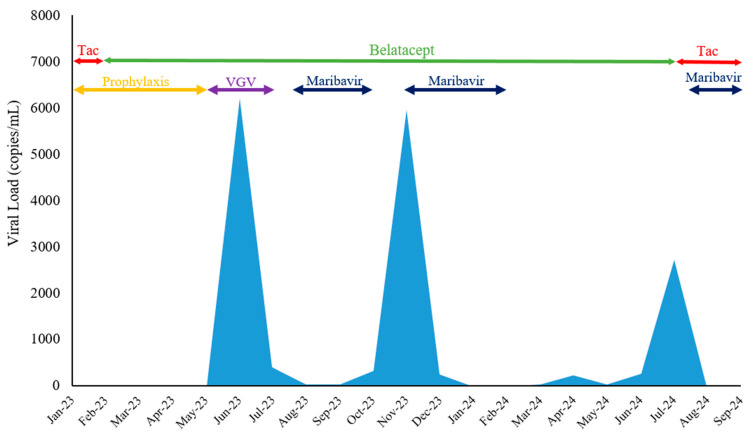
CMV Viral Load Over Time with Corresponding Information on Immunosuppressive Therapy and Antiviral Use. VGV: Valganciclovir, Tac: Tacrolimus.

During her routine follow-up visit in June 2023, leucopenia (white blood cell count of 1.4 × 10^3^ cells/μL) and a drop in platelet count from baseline (165 × 10^3^ cells/μL) was observed. Due to leukopenia and the recent completion of valganciclovir prophylaxis in a D+/R− patient, CMV PCR was checked. CMV quantitative PCR showed 6340 copies/mL (Figure 1). Based on these findings, a diagnosis of CMV infection was made. Mycophenolate mofetil was stopped. The patient began treatment with renally dosed valganciclovir. Weekly CMV levels were monitored for the patient, showing values of 303, 720, and 181 copies/mL over the next three weeks. Due to persistent and undulating viremia and an inability to achieve complete clearance, a decision was made to switch valganciclovir to maribavir (400 mg, PO twice daily). Weekly outpatient CMV PCR were checked and dropped to below the limit of quantification.

After a four-week period, in August 2023, she had no complaints when she presented in the clinic for follow-up. Quantitative PCR for CMV at this visit showed levels below the quantification threshold. WBC and platelet counts improved to 3.3 × 10^3^ cells/μL and 171 × 10^3^ cells/μL, respectively. Her renal profile also showed improvement, with a serum creatinine of 1.69 mg/dL. Maribavir was discontinued after 8 weeks. The patient tolerated maribavir without any untoward side effects. Quantitative PCR for CMV consistently showed levels below the quantification threshold during the next two months. The patient seroconverted during the viremia to a positive test for CMV IgG. Surveillance for BK virus was carried out on a regular basis per our institutional protocol.

Testing in November 2023 showed 6080 copies/mL of CMV. A genotype sequencing-based CMV drug resistance panel was checked and was negative for resistance to ganciclovir, maribavir, cidofovir, and foscarnet (Table 1). Based on the patient’s prior good response to maribavir, maribavir was chosen again for the treatment of recurrent CMV viremia. Treatment with maribavir (400 mg, PO twice daily) resulted in CMV levels dropping below the PCR quantification threshold in the subsequent two months. By February 2024, the test for CMV was negative, and maribavir was discontinued. The second course of maribavir treatment was longer and the patient tolerated it well.

While CMV remained undetectable in the following month, viral reactivation occurred in April 2024, with levels reaching 243 copies/mL. In response, we implemented a modified therapeutic approach by reducing the belatacept infusion dosage to 4 mg/kg every 28 days. This intervention initially achieved viral suppression below the quantification threshold in the subsequent month. However, viral rebound occurred in June 2024, with CMV levels of 268 copies/mL, followed by a further elevation to 2771 copies/mL in July 2024.

In the light of persistent and fluctuating viremia, surrogate tests were performed to assess the patient’s immune response. These included the *Immuknow assay* (which identifies changes in CD4 cell ATP production), *CMV Insight* test (for the evaluation of CMV-specific T-cell immunity), and *Torque Teno Virus (TTV)* test (Torque Teno Virus load measured by quantitative PCR in plasma indicates the intensity of host immunosuppression). The *Immuknow* assay resulted with an ATP level of 144 ng/mL indicative of low immune cell response, and the *CMV Insight* analysis showed CD4 interferon gamma cells at 0.06%, suggesting a high risk of CMV infection. The *TTV* PCR test revealed a level of 7.5 × 10^8^ copies/mL suggestive of an immunosuppressed state with an increased risk of infection. At this point, belatacept was discontinued, and tacrolimus was restarted with a goal trough level of 4–6 ng/mL, along with restarting maribavir. This ultimately led to the clearance of CMV viremia by August 2024, confirmed by two consecutive negative CMV, alongside a new baseline serum creatinine level of 2.2 mg/dL. Maribavir was stopped in September 2024, and viremic clearance was observed, along with follow-up trends in the *CMV Insight* test indicating increasing immunity against CMV and decreasing *TTV* viral load. The patient remained off mycophenolate throughout this course and her DSAs remained negative.

## 3. Discussion

CMV is one of the most prevalent infections affecting transplant recipients, with an incidence of 8% to 32% in renal transplant patients [6]. The precise increase in CMV risk attributable to belatacept remains unclear. In affected patients, CMV can present with a spectrum of clinical manifestations, ranging from asymptomatic viremia to severe, life-threatening infections involving the gastrointestinal system, central nervous system, or the respiratory system. It is typically acquired via donor transmission or reactivation in the recipient, with the highest risk in seronegative recipients (R−) of seropositive donor organs (D+). The lowering of immunosuppression for the management of CMV infection can be complicated by allograft rejection.

The progression of CMV viremia typically diminishes within a few weeks after the initiation of antiviral treatment. In the BENEFIT trial, the incidence of CMV associated with belatacept was comparable to that seen with cyclosporine [5]. Yet recent studies have shown a concerning pattern of persistent CMV manifestations, including viremia and retinitis, in transplant recipients treated with belatacept [2,7]. The duration of CMV in these patients ranges from 3 to 20 weeks, depending on multiple factors, with serostatus being a critical determinant [2,8]. The prolonged course of the disease is hypothesized to result from a loss of CMV-specific T-cell immunity. This loss may be driven by T-cell exhaustion, the inhibition of T-cell activation, increased exposure to viral antigens, and the disruption of the PD-L1/PD-1 signaling pathway [9].

First-line antivirals for CMV viremia, ganciclovir, and valganciclovir require activation by viral UL97 kinase and host kinases, then they inhibit viral DNA polymerase to block replication. Their use is limited by bone marrow toxicity, incomplete suppression, and resistance—especially with low or maintenance dosing. In transplant recipients, resistance to ganciclovir can occur in up to 18% of the cases. While the exact resistance rate in belatacept-treated patients is unknown, it is clinically seen to be higher. This resistance to first-line antivirals typically develops through mutations in either the viral UL97 kinases or UL54 viral DNA polymerase [10].

Traditional second-line treatments for resistant cases include foscarnet and cidofovir. Nevertheless, these drugs not only have an increased likelihood of cross-resistance with ganciclovir but also exhibit significant nephrotoxicity, which limits their use in renal transplant recipients [10]. We chose the relatively novel and oral anti-CMV agent maribavir in our patient due to the incomplete resolution of CMV viremia with valganciclovir. Maribavir received FDA approval in November 2021 for treating CMV infections in transplant recipients. It works differently from first-line agents by directly inhibiting the viral kinase pUL97, which is crucial for viral replication [11]. The advantages of maribavir over other agents are the lack of myelosuppression and renal toxicity. It is usually tolerated well, as it was in our patient. It has shown an impressive efficacy, successfully treating up to 70% of resistant CMV cases [12]. Treatment requires the monitoring of viral levels and continues until two consecutive tests show no detectable viremia.

The complete clearance of viremia with anti-CMV drugs is typically successful, but the recurrence of viremia can still occur. The likelihood of recurrence is higher with valganciclovir/ganciclovir, foscarnet, or cidofovir compared to maribavir, as demonstrated by the SOLSTICE trial [13]. In this trial, recurrence was observed in 26% of patients treated with maribavir, compared to 35.7% in the other group. Although the recurrence rate was lower in the maribavir group, this difference still stands out. We also observed a recurrence of viremia while the patient was off maribavir, but the reintroduction of maribavir achieved complete clearance. In the absence of drug-resistant genetic mutations, this could be attributed to the use of belatacept, which suppresses the ability to mount CMV-specific T-cell immunity once viremia has been cleared. To the best of our knowledge, data on the precise mechanisms behind recurrence following the complete clearance of viremia with maribavir and belatacept is limited. Sequencing CMV DNA to analyze immune-related homologs could offer valuable insights into the pathophysiology of recurrence in such cases.

In cases where belatacept-treated patients fail to achieve complete clearance of CMV viremia despite using new antivirals, three potential treatment strategies can be considered. The first involves switching belatacept to a calcineurin inhibitor while restarting antiviral therapy [14]. In our case, this strategy proved effective. The second strategy involves adding a mTOR inhibitor, such as everolimus or sirolimus, to the treatment regimen alongside belatacept [9]. mTOR inhibitors enhance viral clearance through their immunomodulatory effects, including restoring αβ and γδ T-cell function, promoting interferon-γ production, increasing CMV-specific CD8+ T-cell activity, and decreasing PD-1 and CD85j expression in T cells [15]. The third strategy adopted by some clinicians involves using the same drug that was used for treatment, as secondary prophylaxis, once viremia clearance has been achieved. This approach was not followed in our case because guidance for the maribavir prophylaxis dose is not established yet. We did not use valganciclovir secondary prophylaxis after maribavir use due to the prior failure to achieve viremic clearance with treatment-dose (higher dose) valganciclovir. Moreover, the use of secondary prophylaxis remains controversial in solid organ transplant recipients, with insufficient evidence supporting its sustained benefit in preventing relapse [16].

Given the significant impact of prolonged CMV infections, limiting belatacept use to low-risk transplant recipients may be prudent. Pairing CMV-seronegative donors with seronegative recipients reduced high-risk D+R− transplants from 18.5% to 2.9% without extending wait times, and could support belatacept use in appropriate cases [2,17].

## 4. Conclusions

Our case is unique due to the complex interplay of difficulties in controlling CMV viremia while on belatacept and the pronounced viral rebound following maribavir discontinuation. Renal transplant recipients treated with belatacept are at an increased risk of prolonged and fluctuating CMV viremia. Such infections may be difficult to manage with first-line antivirals, making long-term control challenging even with the use of novel antiviral agents. Clinicians should remain vigilant to atypical CMV infections in such patients, and consider adjustments to the immunosuppression regimen and use of novel anti-CMV agents while closely monitoring the recurrence of viremia.

## Figures and Tables

**Table 1:** CMV Drug Resistance Genotyping Results from the Second Viremic Episode (November 2023).

Test	Result
Maribavir UL97	None Detected
Ganciclovir UL97	None Detected
Ganciclovir UL54	None Detected
Cidofovir UL54	None Detected
Foscarnet UL54	None Detected

## Data Availability

The relevant clinical data from this case report are included in the manuscript, with all data anonymized to the best possible extent.
